# Supplementary material for: Multi‐dataset identification of innovative feature genes and molecular mechanisms in keratoconus
Source: J Cell Mol Med. 2024 Sep 19;28(18):e70079. doi: 10.1111/jcmm.70079 (PMC11412914; doi:10.1111/jcmm.70079)
Supplement: Supplementary file 2 — Material S2. [file JCMM-28-e70079-s002.zip › 7.GSEA analysis/6.SNORA74B/KEGG_GLYOXYLATE_AND_DICARBOXYLATE_METABOLISM.html]

Details for gene set KEGG\_GLYOXYLATE\_AND\_DICARBOXYLATE\_METABOLISM[GSEA]

|  || Dataset | SNORA74B.group.cls#h\_versus\_l |
| Phenotype | group.cls#h\_versus\_l |
| Upregulated in class | h |
| GeneSet | KEGG\_GLYOXYLATE\_AND\_DICARBOXYLATE\_METABOLISM |
| Enrichment Score (ES) | 0.31479853 |
| Normalized Enrichment Score (NES) | 0.8972142 |
| Nominal p-value | 0.56995887 |
| FDR q-value | 1.0 |
| FWER p-Value | 1.0 |
Table: GSEA Results Summary

  

Fig 1: Enrichment plot: KEGG\_GLYOXYLATE\_AND\_DICARBOXYLATE\_METABOLISM      
 Profile of the Running ES Score & Positions of GeneSet Members on the Rank Ordered List

  

| SYMBOL | TITLE | RANK IN GENE LIST | RANK METRIC SCORE | RUNNING ES | CORE ENRICHMENT || 1 | HAO1 | na | 1078 | 0.475 | 0.2340 | Yes |
| 2 | GLYCTK | na | 3587 | 0.274 | 0.3148 | Yes |
| 3 | PGP | na | 14148 | 0.034 | 0.0296 | No |
| 4 | GRHPR | na | 16188 | 0.013 | -0.0221 | No |
| 5 | AFMID | na | 16631 | 0.008 | -0.0303 | No |
| 6 | MDH2 | na | 16764 | 0.007 | -0.0303 | No |
| 7 | CS | na | 17999 | -0.006 | -0.0625 | No |
| 8 | MDH1 | na | 19809 | -0.026 | -0.0999 | No |
| 9 | ACO2 | na | 20093 | -0.029 | -0.0917 | No |
| 10 | HYI | na | 21221 | -0.043 | -0.1004 | No |
| 11 | MTHFD2 | na | 23629 | -0.075 | -0.1279 | No |
| 12 | MTHFD1 | na | 24344 | -0.086 | -0.1004 | No |
| 13 | ACO1 | na | 26383 | -0.125 | -0.0896 | No |
| 14 | MTHFD1L | na | 27391 | -0.149 | -0.0355 | No |
| 15 | MTHFD2L | na | 28624 | -0.183 | 0.0310 | No |
| 16 | HAO2 | na | 30842 | -0.260 | 0.1119 | No |
Table: GSEA details [plain text format]

  

Fig 2: KEGG\_GLYOXYLATE\_AND\_DICARBOXYLATE\_METABOLISM      
 Blue-Pink O' Gram in the Space of the Analyzed GeneSet

  

Fig 3: KEGG\_GLYOXYLATE\_AND\_DICARBOXYLATE\_METABOLISM: Random ES distribution      
 Gene set null distribution of ES for **KEGG\_GLYOXYLATE\_AND\_DICARBOXYLATE\_METABOLISM**
